# A Novel Method of Assessing Floor Friction in Cowsheds and Its Association with Cow Health

**DOI:** 10.3390/ani9040120

**Published:** 2019-03-27

**Authors:** Arvind Sharma, Uttara Kennedy, Clive Phillips

**Affiliations:** Centre for Animal Welfare and Ethics, School of Veterinary Science, The University of Queensland, Gatton Campus 4343, Australia; arvind.sharma@uq.net.au (A.S.); c.phillips@uq.edu.au (C.P.)

**Keywords:** coefficient of friction, floor, cows, housing, welfare, assessment

## Abstract

**Simple Summary:**

The objective of this paper was to test a simple method of measuring the extent of the slipperiness of different types of floors in cow shelters. Excessively slippery floors, which are less abrasive, are known to reduce a cow’s ability to walk, lie down, and get up without slipping and causing injury to herself. A moderate level of friction between the cows’ hooves and the floor is required for cow comfort and prevention of foot disease and injuries. This study developed a rapid method that welfare assessors can use to measure the slipperiness of floors in cowsheds. The data obtained were then validated by comparing them with the various animal-based and resource-based welfare parameters assessed in each shelter. The analyses done in this study show that there is a link between excessively slippery floors and various parameters such as frequency of cleaning the floors, the gradient of floors, wounds on the bodies and joints of the cows, and the presence of dirty hind limbs. All of these ultimately affect the welfare of cows in shelters. These results suggest that this simple measure of floor friction could be useful in cow welfare assessments. Further work on the repeatability of this method is recommended.

**Abstract:**

Measurement of friction of cowshed floors to determine slipperiness potential is important for cow comfort. Existing methods require elaborate equipment and procedures. A quick method for assessment of friction characteristics is proposed. Friction was measured in 54 cattle housing and yard facilities with earth, brick, concrete, and stone floors, and its association with cattle health parameters was investigated through assessment of 30 animals per facility. A 156 g cuboidal wooden block attached to a spring balance was pulled over 3 m, and the coefficient of friction was recorded as the force required to move the block at a constant speed. The coefficient of friction ranged from 0.3 to 0.7 and was lowest for concrete and highest for earth floors. A multivariate analysis found that cows were standing more and could be more easily approached when they were on floors with high friction levels. The proportion of cows with dirty hind limbs declined with increasing friction of the floor, probably reflecting the fact that they felt more confident to stand rather than lie on high friction floors. This simple measure of frictional characteristics of cattle floors offers promise to be included in welfare measures as an indicator of cow welfare.

## 1. Introduction

**Figure 1:**
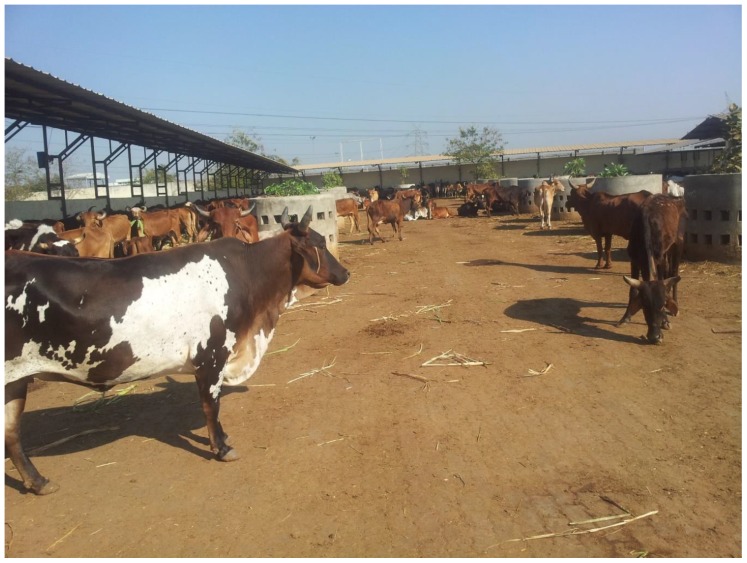
Simple shelter structure for a typical gaushala (photograph by Arvind Sharma).

India has an ancient tradition (from the 2nd century B.C.) of sheltering cows in shelters. These cow shelters (gaushalas) house abandoned, infertile, and non-productive cows. The size of these shelters ranges from fifty to ten thousand cows. The shelters play a significant role in the management of stray cattle in India where cow slaughter is not permitted by law. The cows are sheltered until they die from natural causes. These shelters are managed by philanthropists, trusts, temples, government municipalities, and animal welfare groups. Cow shelters are usually simple traditional structures (Figure 1) with a variety of floor types and little attention to, or routine maintenance of, the floor [1]. The quality of floors of cow sheds is important for cow comfort. Long term wear of the floors renders them smooth and more slippery [2], which may affect getting up, lying down, and walking behaviour. Improper flooring will lead to deprivation or alteration of these behaviours [3]. Contemporary cow welfare assessment studies have assessed types of flooring and bedding [4,5,6], but none of them, to the best of our knowledge, have measured floor slipperiness. The floor surface should be clean and dry for comfortable resting and avoidance of slipping [3]. Floors should allow cows to lie down, rise up, and walk without slipping [7]. Measurable changes in the gait of cows, slipping, falls, and injuries occur due to the absence of adequate friction, usually as a result of poor design or the presence of a slurry of urine and faeces on the floor [8,9,10,11]. Slippery floors restrict the natural locomotion of cows as they are forced to adapt to an unnatural walking environment [12]. Increasing the friction of floors also increases abrasiveness and wear of the hooves of cows, but insufficient abrasiveness leads to overgrowth of their hooves or claws [13]. A judicious tradeoff between the two is required so as to design floors which are neither too abrasive to cause excessive wear of the hooves and joint lesions nor insufficiently abrasive to cause slipping.

Slipperiness has been assessed by measuring friction levels of floors [14]. Floor frictional forces and the reaction of hooves and claws of cows to floor abrasiveness have been studied under laboratory conditions using cow-simulating machines and biological materials in the form of cattle hooves [13,14,15,16]. A coefficient of friction (CoF, the force required to move an object/object mass) is usually measured, which varies inversely with slipperiness of flooring. CoF depends on the hoof, flooring, contact surface between the hoof and the floor, and presence of slurry or other liquids on the floor [11]. Literature has not revealed an easy, on the spot method of assessment that welfare assessors can use to rapidly measure the slipperiness of floors in cowsheds. Floor slipperiness has not been incorporated into welfare assessment protocols for cattle to the best of our knowledge. This could be due to a time-consuming and cumbersome methodology which is difficult to be carried out in routine welfare assessments. Welfare assessments are increasingly common in farms to meet the growing need by members of the public for improved conditions for dairy cows [17]. The objective of this paper was to test a simple method of measuring friction levels of different types of floors found in cow sheds and yards of the cow shelters and validate it with measurements of the characteristics of the buildings and cattle within, in particular, their behaviour and lesions on their limbs. 

There is a lacuna in the scientific literature about the welfare assessment of cows in such shelters in general and assessment of the friction of various types of flooring in these shelters in particular. In this study, we attempted to formulate a novel method of assessing the friction of the floors in cow shelters and then correlate these frictional characteristics with cow health, behaviour, and other relevant measures of welfare, in order to determine if this measure could usefully be added to existing protocols. 

## 2. Materials and Methods 

Fifty-four cow shelters (gaushalas) in six states of India were assessed for animal welfare conditions in the form of 31 resource-based measurements and 28 animal-based measurements (Table 1 and Table 2). A typical cow shelter is an institution in which one or more sheds house the cows. In the cow sheds, there may or may not be an open loafing area present, referred to as the yard, where the cows are able to freely move about, sit, or stand. In case of shelters having multiple sheds and yards (more than two), two representative sheds and adjoining yards were assessed. A total of 86 sheds and 76 yards were assessed in the 54 cow shelters as a part of a welfare assessment protocol. 

A combination of assessment methods (behaviour observations, evaluation of skin alterations indicative of poor comfort levels, and clinical examination) was used to describe the health and welfare status of the cows. For each of these methods, specific indicators that were considered relevant for health and welfare were identified. Indicators which had been described and validated in previous welfare assessment studies conducted in Europe and other western countries in dairy cattle, especially the Welfare Quality^®^ Project protocol, were selected. The 31 resource-based measurements were divided into six main criteria: Housing, specific shed measurements and features, bedding, flooring, watering, and feeding characteristics. The characteristics of the flooring was one of the parameters for assessment. A total of 1620 cows in 54 cow shelters were randomly selected for animal-based measurements, 30/shelter, as recommended following a statistical power analysis. In each cow shelter, 30 cows were sampled as recommended by the power calculation performed for the number of shelters to be sampled and the number of cows to be sampled in each cow shelter. The study was designed to detect an odds ratio of 4 with a power of 0.8 and α = 0.05. A sample size of 30 cows is sufficient to estimate within-herd prevalence with an accepted error of 10% at a 95% level of confidence. Cows were selected randomly by choosing every 3rd cow in the shed or the yard. There was only one observer who carried out the measurements.

Friction levels of the floors of sheds and yards (where present) were assessed using a spring balance measuring 1 kg/10 N [18]. The hook of the balance was attached to a cuboidal wooden block weighing 156 g and being 12.5 × 5.5 × 3.5 cm in length, breadth and height, respectively. The block was gently pulled across the floor, and the minimal frictional force (F) required to move it at a speed of 0.3 m/s over a distance of 3 m was recorded from the scale of the spring balance. The block was pulled at three randomly selected places on each shed and yard floor. The coefficient of friction (CoF) was calculated by the formula:

CoF = weight required to move block ÷ weight of the cuboidal wooden block (CoF = W_SB_/W_B_), (1)

where W_SB_ is the spring balance weight recorded and W_B_ is the block weight. 

Twenty-one sheds had earthen, 19 had brick, four had rock slabs/stones and 42 had concrete based as flooring material (Table 3). Forty-one yards had earthen, 13 had brick, three had stone and 19 had concrete-based floors. The methodology of assessment of the animal- and resource-based measures followed in this study has been elaborated upon in Table 1 and Table 2. 

**Table 2:** Shelter animal-based parameters for assessment of association with coefficient of friction (CoF) of shed floor.

Parameter	Description	Scales and Scores
**Stall Standing Index (SSI)** [19]	Proportion of cows in a stall or shed that were standing	
**Avoidance Distance (AD)** [20]	Cows that were standing at the feeding manger were approached at the front at a rate of one step per second, starting at 2 m from the manger. The distance between the assessor’s hand and the cow’s head was estimated at the moment the cow moved away and turned its head	0—touched 1—0 to 50 cm 2—51 to 100 cm 3—>100 cm
**Rising restrictions** [21]	As a result of shelter facilities	0—Unrestricted (cow is able to rise as if it were in a pasture) 1—Mild restrictions (cow is able to modify standing to rise comfortably as it lunges sideways and not forwards) 2—Cow takes time to rise and hits shed fixtures or fittings while rising 3—Dog-sitting posture adopted while standing or makes multiple attempts before able to rise.
**Rising behaviour** [22,23]	All cows lying in the shelter were coaxed to get up with use of a minimum amount of force. If the presence of the assessor did not evoke rising they were given one or two gentle slaps on the back, followed by a break of 5 s, then more slaps with slightly more force if required, up to a maximum of 30 s	1—Normal (smooth and a normal sequence of rising behaviour) 2—Easy but slightly interfered (smooth movement with a slight twisting of the head but with a normal sequence of rising process) 3—Uneasy with effort (sudden movement and difficulty in rising with awkward twisting of the head and neck but following a normal sequential rising process) 4—Abnormal (abnormal sequence of rising event) 5—Refused to get up
**Body Condition Score (BCS)** [24]	A cow with a score of ≤1.25 was considered emaciated, 1.5–2 thin, 2.25–3.75 normal and 4 or more obese Visual examination	1 to 5 with increments of 0.25.
**Lactation**		0—Non-lactating 1—Lactating
**Lameness** [25]	1 to 5 scale Visual examination	1—not lame (smooth and fluid movement) 2—mildly lame but not observable easily (an imperfect gait but able to freely move with a mildly arched back) 3—moderately lame (able to move but not freely, with an arched back) 4—lame, with inability to move freely with an asymmetrical gait and abnormal head movement 5—severely lame (severely restricted in movement, requiring considerable encouragement to move, and a severely arched back)
**Claw overgrowth** [21]	Visual examination	0—Normal claws 1—Mild claw overgrowth 2—Moderate claw overgrowth 3—Severe claw overgrowth
**Hock joint swellings** [26]	Visual examination	1—mild swollen joint 2—medium swollen joint, 3—severely swollen joint
**Hock joint hair loss and ulceration** [26]	Visual examination	0—no hair loss or ulceration 1—mild hair loss or ulceration <2 cm^2^ 2—medium hair loss or ulceration (approx. 2.5 cm^2^) 3—severe hair loss or ulceration >2.5 cm^2^
**Carpal joint injuries** [27]	Visual examination	0—no skin change 1—hairless 2—swollen 3—wounded
**Dirtiness of the hind limbs, udder, and flanks** [26]	By visual inspection of the cows from a randomly chosen side (left or right) and from behind	1—no dirtiness 2—mildly dirty (small soiled areas of dirtiness with no thick scabs) 3—medium dirtiness (large soiled areas but with <1 cm thick scabs of dung) 4—severely dirty (large soiled areas with >1 cm thick dung scabs)
**Body coat condition** [21]	Visual examination	1—dull and short 2—shiny and short 3—dull and hairy
**Ectoparasitism** [28]	Visual examination	1—Absence of ectoparasites 2—Mild infestation—no lesions (not easily visible by naked eye but on tactile perception in the neck region) 3—Moderate—mild infestation (visually observable ectoparasites or immature forms or eggs in the neck, groin, peri rectal, tail root and switch regions) 4—Severe—Visual observation of mature ectoparasites all over the body, especially regions mentioned in score 3
**Skin lesions** [29]	Visual examination	0—normal (no apparent lesions) 1—mild hair loss (≤2 cm^2^) 2—moderate (>2 cm^2^ hair loss and inflamed skin) 3—severe (a large >4 cm^2^ area of hair loss with extensive skin inflammation and breakage)
**Skin tenting time** [30]	Visual examination by skin pinch of the cervical region of the neck	1—≤2 s 2—>2 s 3—≥6 s
**Teat and udder condition**	Visual inspection	1—Normal teats and udder 2—Dry udder and teats 3—Teat cracks 4—Warts on teats and udder 5—Acute lesions on the teats and udder, 6—Chronic lesions on teats and udder
**Rumen fill score** [31]	Visually, by standing behind the cow on the left side and observing the left para lumbar fossa between the last rib, the lumbar transverse processes, and the hip bone	1—the para lumbar fossa is empty, presenting a rectangular cavity that is more than a hand’s width behind the last rib and a hand’s width under the lumbar transversal processes 2—the para lumbar fossa forms a triangular cavity with a width about the size of a hand behind the last rib, but less than this under the lumbar transverse processes 3—the para lumbar fossa forms a cavity less than a hand’s width behind the last rib and about a hand’s width vertically downwards from the lumbar transverse processes and then bulges out 4—the para lumbar fossa skin covers the area behind the last rib and arches immediately outside below the lumbar transverse processes due to a bloated rumen 5—the rumen is distended and almost fills up the para lumbar fossa; the last rib and the lumbar transverse processes are not visible.
**Diarrhoea** [32]	Visual examination	0—absent, 1—present
**Hampered respiration** [32]	Visual examination	0—absent, 1—present
**Nasal discharge** [32]	Visual examination	0—absent, 1—present
**Ocular lesions** [32]	Visual examination	0—absent, 1—present
**General demeanor** [33]	Visual examination	0—docile, 1—aggressive

**Table 3:** Shed coefficients of flooring for four types of flooring in cow shelters (*n* = 86).

Type of Shed Flooring	Number	Median Coefficient of Friction	IQR
**Earth**	21	0.67	0.075
**Brick**	19	0.57	0.171
**Rock/stone**	4	0.39	0.246
**Concrete based**	42	0.29	0.163

Interquartile range (IQR).

### 2.1. Statistical Analysis:

All the analyses were run at 5% assumed level of significance using a computerised statistics software (Minitab^®^ version 17.1.0, Minitab Inc., State College, PA, USA). Each set of observations in a facility was assumed to be independent of all others. The differences between the coefficients of friction of different types of floors were calculated by the Mood’s Median test because residuals after a general linear model were not normally distributed. The 54 shelters were considered as a fixed factor. The coefficients were taken as a continuous response variable.

Overlap in factors associated with the coefficients of friction was initially identified by a Principal Components Analysis (PCA) of the animal-based as well as the resource-based parameters. As a result, values for % of dung in the passageways and lying areas were combined. The variables were then subjected to a univariate analysis with the coefficient of friction of the flooring, using Spearman’s Rank Correlations because several variables were not normally distributed. The variables having a correlation with the coefficient of friction at a *p*-value of less than or equal to 0.05 were retained and subjected to multivariate analysis using a general linear model employing a backward elimination stepwise process to identify the association of risk factors with the coefficient of flooring. Alpha to remove variables was set at 0.25. There were only four shelters that had stone/rock floors, and hence they were not included in the model. Although we determined the CoF of the flooring of sheds and yards, in the multivariate analysis, we used the shed CoF only because many shelters did not have yards. Variance inflation factors were inspected to ensure low levels of collinearity between variables. Residuals were tested for normality by the Anderson–Darling test.

## 3. Results

The overall median floor CoF was 0.43 ± 0.194 SD. The potential range of CoF in our study was from 0 to 1, and the actual range was 0.61, from 0.11 minimum value to 0.72 maximum value. The median CoF was higher for earth and brick floors than stone and concrete (Table 3) (chi-square value = 52.78, df = 3, *p*-value < 0.001).

The descriptive statistics of the animal-based and resource-based parameters used in the linear model are presented in Table 4 and Table 5, respectively. Spearman’s rank order correlation between the coefficient of friction of the shelter floors (continuous variable) and ordinal and continuous variables of the resource- and animal-based measures demonstrated significant correlations in both categories of variables (Table 6). CoF was positively related to the % of faeces in the lying areas and passages, and it was increased in sheds that were not cleaned. In sheds that were cleaned, it was negatively correlated with the frequency of scraping. It was also positively correlated with the gradient of the passages. In terms of animal-based measures, a negative correlation with the stall standing index indicated that floors with a high CoF had fewer cows standing. Floors with a high CoF had cows with more body hair loss and body lesions, but fewer swellings and ulceration of the hock joints and injuries to the carpal joints.

**Figure 2:**
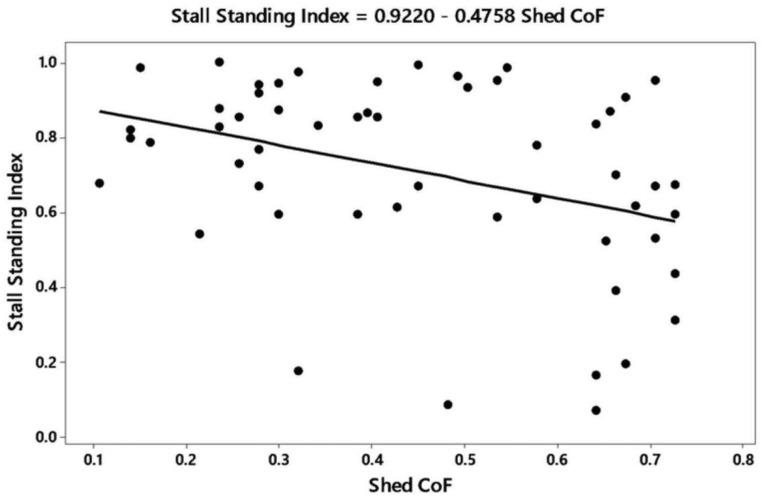
Relationship between the proportion of cows standing (stall standing index) and coefficient of friction (CoF) of shed floor.

**Figure 3:**
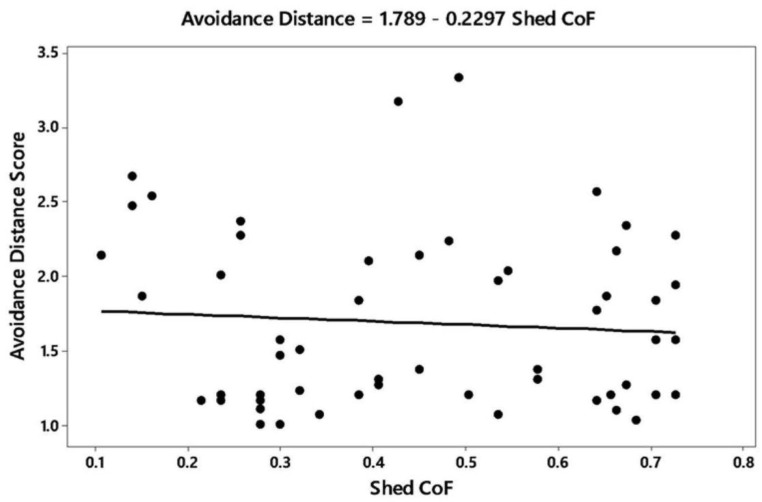
Relationship between the avoidance distance score and coefficient of friction (CoF) of shed floor.

**Figure 4:**
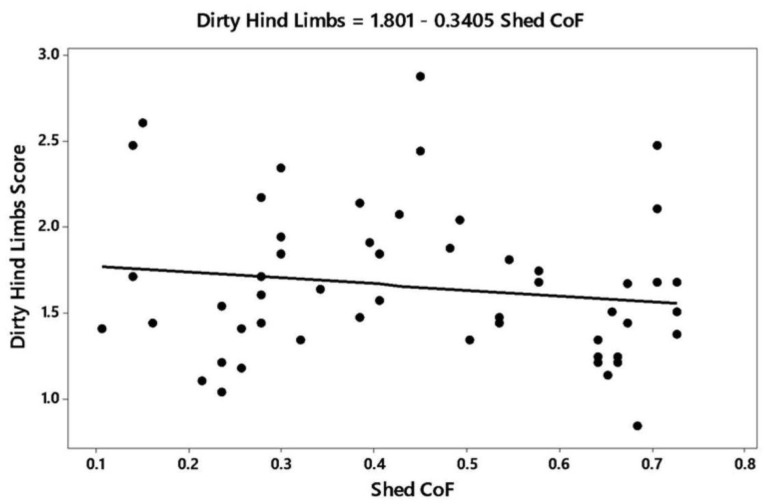
Scatter plot showing relationship between dirty hind limbs score and coefficient of friction (CoF) of shed floor.

In the multivariate analysis of CoF with animal and shed variables, there were four variables significantly related to the coefficient of friction (r^2^ adjusted = 82.8; residuals of the model were normally distributed): Stall standing index (*p* = 0.01), avoidance distance (*p* = 0.04), dirty hind limbs (*p* = 0.03), and shed flooring (*p* < 0.001). For the stall standing index, more cattle were standing as CoF decreased (Figure 2). Avoidance distance decreased as CoF increased (Figure 3), and the proportion of cows with dirty hind limbs decreased with CoF (Figure 4). The relationship was described by the equation:

Shed flooring CoF = c − 0.157 Stall Standing Index (±0.0577, *p* = 0.01) − 0.0649 Avoidance Distance Score (±0.0299, *p* = 0.04) + 0.0861 Dirty Hind Limbs Score (±0.0377, *p* = 0.03), (2)

where c is the intercept, which for earthen floors was 0.812 and for brick floors was 0.736, relative to concrete floors which was 0.442; *p* < 0.001 and *p* = 0.002, respectively.

Other variables that were not significant (*p* > 0.05) but were initially included in the regression equation were floor scraping frequency (coefficient —0.020 (±0.0147), *p* = 0.18), body hair loss (coefficient —0.043 (±0.0272), *p* value 0.13) and nasal discharge (coefficient + 0.213 (±0.124), *p* = 0.09).

## 4. Discussion

The objective of developing a method of measuring CoF that related to resource- and animal-based characteristics in different types of cattle accommodation was achieved. Through the measurements of CoF, the results indicated an interactive relationship between the environment in cow shelters and the reaction of cows to that environment, quantified through the measurement of various cow- and resource-based measures. The coefficient of friction of flooring in this study ranged from 0.3 to 0.7 across four types of shelter flooring (earthen, brick, stone, and concrete), which is a broader range than that calculated by Penev et al. [34]. The higher the value of the coefficient of friction of a floor is, the lower the probability of slipping is [9]. The lower end of the range calculated in our study was below the critical point of 0.4 to avoid slipping, as suggested by Phillips and Morris [9] and Van der Tol et al. [11]. However, this is not surprising as the Van der Tol et al. [11] study evaluated only two types of floors: Concrete and rubber matting floors. There is a tendency of cows to walk quickly in short steps on floors with lower friction, while they walk slowly with longer steps on floors with higher friction [9].

CoF was highest for earthen floors, intermediate for brick floors, and much reduced for concrete floors. The small number of stone floors appeared to be most similar to concrete floors in frictional characteristics. Concrete floors wear smooth over time, and even if they are grooved with a diamond cutter [35], they still wear down with constant traffic of cows on the floor.

The negative association of the frequency of scraping the shelter floors with CoF was demonstrated by the finding that CoF was higher in floors that had faeces in lying areas as well as passages. This is similar to the increase in frictional characteristics of the floor that was previously found for floors with an aggregate embedded [9], for which it was suggested that the greater CoF on floors with aggregate presented a vertical impediment to the motion of the block.

The method used in this study attempted to mimic the sliding frictional aspect of the cow’s movement on floors, as this movement truly reflects the risk of slipping. The presence of only urine on the floors of the sheds and passages did not significantly affect the CoF. This partially corroborates the studies of Phillips and Morris [8] who found no changes in the gait of cows when the floor was wet, though the limb movement angles, as well as patterns, were affected. However, the presence of faeces increased the CoF of the floors in our study, as has been previously reported [9].

The stall standing index (SSI), devised by Cook et al. [36], is one of the indices for the assessment of comfort levels of cows in a stall, or in shelters in our case. The negative correlation between the CoF and SSI in our study suggests that with an increase in CoF, the cows were less likely to be standing and more likely to be lying down, reflecting greater comfort levels on floors with higher friction. Slipping whilst standing is more likely at low friction levels [10,37,38]. Floor bedding may work in a similar way to faeces on the floor, providing resistance to horizontal motion.

The avoidance distance (AD) measure is used to quantify the human–animal relationship and to assess an animal’s fear of humans [39]. The model revealed a negative relationship between AD and CoF, thus high friction floors had cows that would permit a very close approach by a researcher. Cows will potentially be less nervous and more comfortable on floors that permit safe movement, and there is an absence of slipping. Flooring with a low CoF and increased slipperiness impedes the natural behaviour of cows [40]. A reassessment of the design of free stalls for cows has been recommended if SSI is more than 0.2 [41]. There is a dilemma that cows need to be active and walk, which can only be done when the cow is standing, but after being active they need to rest. A simple measure of the proportion of cows standing is not sufficient to understand the complexities of cows’ needs and further work is needed in this area to develop simple measures that measure the cows’ needs better.

A possible negative correlation between the CoF and body hair loss was suggested (*p* = 0.13) but was not significant. If confirmed, it can be explained by the frequent slipping of the cows causing injuries and hair coats getting contaminated with dung. These might lead to loss of hair on the body. It has been suggested that lesions and swellings in the body of dairy cows are influenced by the quality of flooring in the passages and stalls and the presence or absence of bedding on it [6,42]. Norberg [43] further proved that floor surface influences the cleanliness of cows and stalls. The presence of dung on various body parts might lead to skin irritation and subsequent hair loss [44].

The correlation of CoF with dirty hind limbs may be because some cows were lying in passageways with excreta. Often, it is only a minority of cows that engage in this behaviour if suitable free stalls/cubicles are provided [45]. For high friction floors, the proportion of dirty hind limbs declined with the CoF, which would be expected if cows felt confident to stand more on these floors. This finding could also reflect the lack of cleaning of sheds with high CoF, which was found in the univariate analysis. The relationships confirm that hygiene levels and lesions on the body of cows reflect the design of the facility, which includes flooring [40,46].

The possible correlation between the CoF and nasal discharge in the cows, although only a trend (*p* = 0.09), if confirmed in other studies could be due to high CoF floors having more faeces, which produces ammonia. This leads to irritation of the nasal mucous membranes. A correlation between floor slipperiness and ammonia emissions in cow housing has previously been demonstrated [47]. The results of the multivariate analysis revealed that lower CoF friction renders the flooring slippery due to which the cows prefer to stand instead of slipping and falling down as shown by the increase in the SSI. Moreover, the majority of our shelter floors were concrete ones and studies have shown that cows prefer to remain standing for longer on such floors [48], which supports the higher SSI in our study. The cows might have felt more comfortable standing and walking on floors having higher CoF as their feet have a better grip with the floor and thus their AD was lower than cows on lower CoF floors. Cows have a natural predisposition to walk for about an hour a day, at 3–4 km/h, hence many cows in shelters are likely to have an unfulfilled urge for activity [49]. Asymmetry in the gait of dairy cows has been found to be less in floors that have low levels of slipperiness [50]. The reduced gait asymmetry results in an absence of nervousness and walking discomfort [50].

The dirtiness of the hind limbs decreased with increasing CoF probably because the cows slipped less, and even because the cows were more confident in their walking and less likely to knock into objects or other cows. These suppositions would need to be confirmed experimentally.

The inclusion of animal-based health indicators in this study has been validated by their significant correlations with the floor coefficient of friction. Other studies have demonstrated correlations between floor characteristics and animal health; for example, it has been revealed that cows have reduced immunity on concrete floors compared to rubber floors, which the authors attributed to increased stress [51].

No effects of the coefficient of friction on other animal-based welfare indicators were observed. The reason could be the variability in flooring in the cow shelters. The fact that the majority of the animal-based indicators were not normally distributed supports this observation. We describe the characteristics of the gaushalas in detail in a separate paper [52].

Future studies could include animal behaviour in more detail, e.g., confidence in walking, knocking into objects or cows, and even the ability of cows to balance on three legs to scratch themselves which would be expected to increase at high friction levels. Subtle indicators of cow comfort on floors of different textures and friction levels are therefore warranted. It would also be interesting to correlate block performance with hooves obtained from an abattoir, as used previously [15]. Uneven hooves may enmesh with the floor better, leading to increased CoF. Theoretically, the size of the block will not alter friction, but different sizes may enmesh with the floor surface, depending on its variability in surface smoothness, to a variable degree, leading to differences in CoF.

## 5. Conclusions

Flooring is a vital component of welfare for a cow facility and has been included in most cow welfare assessments in different parts of the world. The purpose of developing this method of measurement of friction of various floors was to provide an easy, affordable, and quick method of assessment. Univariate analysis yielded a correlation of the CoF with shed flooring, bedding, cleanliness of the cow sheds, frequency of cleaning of the floors, gradient of the floors, lesions on the tarsal joints of the cows, lesions on carpal joints, and on the body. The multivariate analysis led to the identification of confirmed correlates with the friction of floors, which were the type of flooring, the proportion of cows standing, the avoidance distance of the cows, and the presence of dirty hind limbs. This analysis has validated our hypothesis that CoF does affect the welfare of cows in shelters. The results of this study suggest that this simple measure of floor coefficient of friction could be a useful measure in cow welfare assessments. Further work on the validity, repeatability, and reproducibility of this method for the measurement of slipperiness of flooring of cowsheds is recommended.

## Figures and Tables

**Table 1:** Shelter housing parameters for assessment of floor characteristics.

Criterion	Parameter	Measurement Description
**Flooring**	Type of flooring	Earth, Brick, Concrete, Stone/Rock
**Bedding**	Type and thickness of bedding	Type, thickness of bedding (in cm) (if any)
**Cleanliness**	Presence of faeces in the lying areas and passages separately	Visual estimation of % of faeces in the passages and lying areas *
	Presence of urine in the lying areas and passages	Visual estimation of % of urine in the passages and lying areas
	Water pooling in the lying areas	Present/absent
**Space allowance**	Area/cow (m^2^)	Area of the shed ÷ Number of cows in the shed
**Floor gradient**	Floor gradient of the lying areas and passages	Ratio of incline to length (as measured by inclinometer)

* For estimation of cleanliness levels, each shed floor was divided into four quadrants; % of dung in each quadrant was estimated visually and an average was taken for the entire floor. Pilot trials were conducted initially to standardise each resource- and animal-based parameter.

**Table 4:** Descriptive Statistics for animal-based measures in the cow shelters measured on ordinal as well as continuous scales.

Parameter	Mean/Median *	Standard Deviation	First Quartile Q*_1_	Third Quartile Q*_3_	Interquartile Range IQR *	*p* Value (>0.05 = Normally Distributed Data)
Cow age (years)	11.0	2.022				0.37
Lactating cow %	0.03 *		0	0.2	0.2	
Temperament, log_10_ of values	0.41 (2.61)	0.068				0.24
Stall Standing Index (SSI)	0.77 *	0.25	0.59	1.0	0.31	
Avoidance Distance (AD) score (Scale 1–4)	1.53 *		1.20	2.13	0.93	
Body condition score (Scale 1–5)	2.69	0.37				0.27
Lameness score (Scale 1–5)	1.13 *		1.05	1.27	0.22	
Claw overgrowth score (Scale 0–3)	0.61 *		0.23	0.90	0.67	
Hock joint swelling score (Scale 0–3)	1.64 *		0.23	2.23	0.44	
Hock joint hair loss score (Scale 0–3)	1.05	0.30				0.22
Hock joint ulceration score (Scale 0–3)	0.59	0.39				0.16
Lateral hock joint swelling score (Scale 0–3)	0.87	0.41				0.88
Lateral joint hair loss score (Scale 0–3)	0.27 *		0	1.30	0.26	
Lateral joint ulceration score (Scale 0–3)	0.11 *		0	1.13	0.20	
Carpal joint injuries score (Scale 0–3)	0.78	0.45				0.18
Dirty hind limbs score ** (Scale 0–3)	0.21 ** (1.59)	0.11				0.64
Dirty udder score (Scale 0–3)	1.27	0.56				0.90
Dirty flanks score (Scale 0–3)	1.24	0.57				0.95
Body hair loss score (Scale 0–3)	0.76 *		0.066	2.03	1.04	
Coat condition score (Scale 1–3)	1.54	0.298				0.08
Ectoparasitism score (Scale 0–3)	1.51 *		0.97	3.27		
Skin tenting time score (Scale 0–4)	0.03 *		0	0.83		
Teat condition score (Scale 0–5)	1.0 *		0.92	1.00	0.075	
Neck lesions score (Scale 1–5)	1.03 *		1.0	1.10	0.1	
Ocular lesions score (Scale 0–1)	0.06 *		0.033	0.13	0.1	
Nasal discharge score (Scale 0–1)	0.05 *		0.000	0.14	0.14	
Rumen fill score (Scale 1–5)	3.68 *		3.19	3.90	0.71	
Diarrhoea score (Scale 0–1)	0 *		0	0.033	0.033	

* Data not normally distributed; ** Log_10_ transformed.

**Table 5:** Median, first quartile (Q_1_), third quartile (Q_3_), and interquartile range (IQR) values for not normally distributed and mean, standard deviation (SD), and *p*-values for normally distributed data, for resource-based parameters for cows in shelters.

Variable	Median/Mean *	SD	First Quartile Q_1_	Third Quartile Q_3_	Interquartile Range (IQR)	* *p*-Value (>0.05 = Normal Distribution)
Area/loose housed cow (m^2^)	2.73		1.56	3.63	2.07	
Area/tethered cow (m^2^)	4.50 *	2.75				0.04
Shed eave height (m)	3.80		2.99	5.34	2.35	
Shed luminosity (lux)	582		89	1036	946	
Shed noise level (dB)	27.7		21.3	37.2	15.8	
Yard noise level (dB)	25.3		20.33	33.00	12.7	
Shed dry bulb temperature (°C)	29.5		27.2	32.8	5.6	
Shed humidity (%)	34.0		24.7	45.2	20.5	
CoF of shed passage floors	0.43		0.27	0.65	0.37	
CoF of yard passage floors	0.64		0.34	0.68	0.34	
Gradient of shed lying areas	1.46		0.96	2.2	1.23	
Gradient of shed passages	2.36		1.27	3.52	2.24	
Gradient of yard floors	1.51		1.13	2.43	1.30	
Dung on shed lying areas (% of area)	15		5	40	35.	
Dung on shed passages (% of area)	10		5	42.5	37.5	
Dung on yards (% of area)	20		10	40	30	
Roughage/cow (kg fresh)	1.25 ** (17.66 *)	0.168				0.06

* Mean; ** Log_10_ transformed.

**Table 6:** Spearman’s rank correlations between coefficient of friction of shelter flooring and resource- and animal-based variables with *p*-values ≤ 0.05.

Variables	Correlation Co-Efficient	*p* Value
**Resource-based**		
Shed flooring type	−0.75	<0.001
Shed % of faeces in lying area	0.37	0.005
Shed % of faeces in passages	0.320	0.02
Shed cleaning (absence 0, presence 1)	−0.29	0.03
Scraping frequency of sheds	−0.42	0.001
Shed average gradient of passages	0.29	0.03
**Animal-based**		
Stall Standing Index (SSI)	−0.33	0.01
Body hair loss	0.31	0.02
Hock joint swellings	−0.32	0.02
Hock joint ulceration	−0.27	0.05
Carpal joint injuries	−0.31	0.02
Lesions on the body	0.30	0.02
